# Haematological, clinical–chemical and immunological consequences of feeding *Fusarium* toxin contaminated diets to early lactating dairy cows

**DOI:** 10.1007/s12550-016-0258-6

**Published:** 2016-11-09

**Authors:** Sven Dänicke, Janine Winkler, Ulrich Meyer, Jana Frahm, Susanne Kersten

**Affiliations:** Institute of Animal Nutrition, Friedrich-Loeffler-Institute (FLI), Federal Research Institute for Animal Health (FLI), Braunschweig, Germany

**Keywords:** Dairy cow, Haematology, Clinical chemistry, Immune system, Mycotoxins, Deoxynivalenol, Zearalenone

## Abstract

Dairy cows experience a negative energy balance at the onset of lactation which results in an enhanced vulnerability for infectious diseases. Any dietary imbalances, including *Fusarium* toxin contamination, might therefore exacerbate this situation. The aim of the present investigations was to study the effects of increasing dietary concentrations of deoxynivalenol (DON) and zearalenone (ZEN) on clinical-chemical, haematological and immunological traits up to week 14 of lactation. For this purpose, ten cows each were assigned to a control group (CON; 0.02 mg ZEN and 0.06 mg DON per kg diet at 88 % DM), toxin level 1 (TOX-1; 0.29 mg ZEN and 2.31 mg DON per kg diet at 88 % DM) and toxin level 2 (TOX-2; 0.58 mg ZEN and 4.61 mg DON per kg diet at 88 % DM). The measured values of most parameters were affected by parturition but only a few of them were further modified by dietary treatment. For example, the time-dependent decrease in haemoglobin concentration, haematocrit and erythrocyte counts occurred at a significantly higher level for group TOX-2 while a serum glucose increase was missing in this group. Proportions of CD4+ and CD8+ cells decreased significantly over time solely in group TOX-2 while the CD4+/CD8+ ratio remained uninfluenced. Ability of granulocytes to mount an oxidative burst tended to increase at the end of the study in groups TOX-1 and TOX-2 while the opposite was observed in group CON. The results of this time-limited study indicate that feeding of *Fusarium*-toxin contaminated diets in early lactation affects health related parameters without compromising milking performance. However, long-term consequences of the observed effects on health need to be addressed in further studies.

## Introduction

Dairy cows experience a negative energy balance (NEB) during the first weeks *post partum* due to the onset of lactation and the insufficient energy intake. NEB is thought to be closely associated to an immune suppression and consequently to an increased susceptibility to infectious diseases. Thus, special care should be devoted to the avoidance of feed contamination by substances which might potentially further compromise the immune system. Mycotoxins are such substances with deoxynivalenol (DON) and zearalenone (ZEN) being important feed contaminants in terms of frequency of occurrence and level of contamination (Döll and Dänicke 2011). Although ruminants are regarded as quite resistant to both toxins, mainly due to the ruminal toxin metabolism of DON and ZEN resulting in less toxic or active derivatives (de-epoxy-DON/de-DON) or such with equal or lower (β-zearalenol [ZEL]), and to a less extent with higher activity (α-ZEL) than the parent toxins (Dänicke and Brezina 2013, Dänicke and Winkler 2015), the impact of feeding diets contaminated with DON and ZEN in a physiological state energetically critical for the cow on health status and immune system was addressed only in a few studies (Kinoshita et al. 2015, Keese et al. 2008a, b). As immune responses require additionally energy it seems to be reasonably to hypothesise that the energy status of the cow is related to health- and immune-traits.

Therefore, one aim of the present investigations was to study the effects of feeding diets with increasing concentrations of DON and ZEN, covering the range from very low levels of DON and ZEN to the critical level of 5 mg DON and 0.5 mg ZEN per kg diet at a reference dry matter (DM) content of 88 % (European Commission 2006), to early lactating dairy cows on clinical–chemical, haematological and immunological traits. These data complete the results on the general effects of dietary treatments on performance traits and mycotoxin residue levels in various matrices as reported recently (Winkler et al. 2014a, Winkler et al. 2014b, Winkler et al. 2015a, Winkler et al. 2015b).The second aim was to relate the health- and immune-related characteristics investigated in the present study to the performance, the energy status and to mycotoxin residue levels reported recently (Winkler et al. 2014a) in order to obtain a more comprehensive view on the consequences of feeding diets contaminated with DON and ZEN on the physiological sensitive early lactating dairy cow.

## Material and methods

### Experimental design, diets and procedures

The experiment and procedures were performed at the experimental station of the Institute of Animal Nutrition Braunschweig in compliance with the European Community regulations concerning the protection of experimental animals and was approved by the Lower Saxony State Office for Consumer Protection and Food Safety (LAVES), Germany.

Samples for the present investigations were obtained from a feeding experiment with dairy cows recently described in detail elsewhere (Winkler et al. 2014a). Briefly, the experimental design consisted of a total of 30 German Holstein cows assigned to three feeding groups with ten cows (4 primi- and 6 pluriparous cows) in each of the following: control group (CON; 0.02 mg ZEN and 0.06 mg DON per kg diet at 88 % DM), toxin level 1 (TOX-1; 0.29 mg ZEN and 2.31 mg DON per kg diet at 88 % DM) and toxin level 2 (TOX-2; 0.58 mg ZEN and 4.61 mg DON per kg diet at 88 % DM).

Diets were offered as a total mixed ration (TMR) for *ad libitum* consumption in self-feeding stations (Type RIC, Insentec, B.V., Marknesse, the Netherlands) from day 7 *post partum* for the next 13 weeks. TMR consisted of 50 % grass silage and 50 % concentrate on DM basis (Table 1). The concentrate portion of the diets was used as the carrier for the mycotoxins whereby *Fusarium* contaminated maize kernels and cobs obtained from an artificial inoculation with *Fusarium graminearum* (for details see Rempe et al. 2013) were admixed to achieve the targeted contamination levels. Cows were kept in pens equipped with slatted floors and cubicles with rubber mattresses and wood litter. Animals were equipped with ear transponders enabling automatic recording of feed and water intake, body weight and milking performance.

**Table 1:** Components, energy content and composition of the concentrates (*n* = 2) and the grass silage (data from Winkler et al. 2014a)

	Concentrate		Grass silage
	CON	FUS	
Components [%]			
Dried beet pulp	30.7	30.7	
Barley	21.0	20.0	
Rapeseed extraction meal	20.0	20.0	
Maize	20.0		
Fusarium toxin contaminated maize		20.0	
Soybean extraction meal	6.5	6.5	
Fusarium toxin contaminated maize cobs		1.0	
Mineral feed^a^	1.8	1.8	
DM [%]	88.0	88.0	42.0
NEL [MJ/kg DM]	7.70	7.60	5.89
Nutrient composition[g/kg DM]			
Crude ash	61	61	92
Crude protein	184	186	133
Crude fat	34	29	35
Crude fibre	107	107	279
Acid detergent fibre	141	145	293
Neutral detergent fibre	282	286	496
Calculated mycotoxin composition [mg/kg DM]			
DON	0.00	9.80	
ZEN	0.00	1.00	
Analysed mycotoxin composition [mg/kg DM]			
DON	0.13	10.47	0.00
ZEN	0.04	1.31	0.01

*DM* dry matter, *NEL* net energy lactation

^a^Per kg mineral feed: 170 g Ca; 120 g Na; 50 g P; 45 g Mg; 6 g Zn; 5 g Mn; 1.3 g Cu; 120 mg I; 40 mg Se; 35 mg Co; 800,000 IU vitamin A; 100,000 IU vitamin D_3_; 4 g vitamin E

A total of four blood samples were collected from each cow in the course of the experiment; a “zero” sample before experimental diets were introduced in week 1, and in weeks 3, 10 and 14 of experiment by puncturing of a *Vena jugularis externa*.

## Sample preparation and analyses

### Haematology and clinical chemistry

EDTA blood was used fresh on the day of blood collection while heparinized plasma and serum samples were centrifuged at 2123×*g* for 15 min at 15 °C (Heraeus Varifuge® 3.0R) and frozen in portions (−80 °C) until analysis.

Red and white blood cell counts were determined in EDTA blood samples using the automatic haematology analyser Celltac-α (MEK 6450, Nihon Kohden Corporation, Tokyo, Japan).

Non-esterified fatty acids (NEFA), beta-hydroxy butyrate (BHB), glucose, albumin, total protein, triglycerides, cholesterol, urea, total bilirubin, aspartate aminotransferase (ASAT), glutamate dehydrogenase (GLDH) and gamma-glutamyltransferase (GGT) were determined in serum samples by using an automatic clinical chemistry analyser (Eurolyser CCA180, Eurolab, Hallein, Austria).

### Flow cytometry

Capability of granulocytes and PBMC to generate reactive oxygen species (ROS) was investigated by flow cytometry (FACS Canto II, BD Biosciences, San Jose, USA). The method is based on the intracellular conversion of the non-fluorescent dye dihydrorhodamine 123 (DHR) to the fluorescent rhodamine 123 as outlined elsewhere (Stelter et al. 2013). In brief, tetradecanoyl-12, 13-phorbol acetate (TPA) served as a positive control based on its ability to stimulate NADPH-oxidase activity.

Based on forward and side scatter measurements, granulocytes and PBMCs were gated according to their size and granularity. At least 10,000 cells were evaluated. Results were expressed as percentages of ROS producing granulocytes of total granulocytes, either unstimulated (basal) or TPA stimulated, as mean fluorescence intensity (MFI) of total or ROS positive granulocytes, as ratio between the MFI of TPA-stimulated and unstimulated ROS positive granulocytes and as total peripheral blood granulocytes exhibiting a basal ROS production. The latter corresponds to the product of total blood granulocyte concentration multiplied with the proportion of basal ROS producing granulocytes.

T cells were phenotyped in EDTA blood samples by staining with monoclonal antibodies (mAbs) for CD4 (Mouse anti bovine CD4: FITC), CD8 (Mouse anti bovine CD8: RPE) or the corresponding isotype controls (Mouse IgG2a negative control: RPE, Mouse IgG2b negative control: FITC) for 30 min at room temperature. Used antibodies were purchased from AbD serotec, Bio-Rad laboratories, Puchheim, Germany.

The samples were centrifuged in hepes buffered saline (HBS) after erythrocyte lysis using lysis buffer (BD Pharm Lyse™, BD Biosciences, San Jose, USA) and finally screened for CD4+ and CD8+ T cells by setting an acquisition gate for the lymphocyte population based on their side and forward scattering properties using a BD FACS Canto II. A minimum of 10,000 lymphocytes were counted and stored in list mode data files. The spillover of both fluorochromes (FITC, PE) was compensated using the BD FACS Diva Software (BD Biosciences, San Jose, USA). Results were reported as percentage of CD4+ and CD8+ cells of the total lymphocytes, as the ratio between both surface phenotypes and as absolute concentration in blood by using lymphocyte counts measured by using Celltac-α (see above).

### Statistics

Data were evaluated in two steps. First, the variance of the data was examined by using procedure MIXED (Software package, version 9.1, SAS-Institute-Inc. 2003, Cary, NC, USA) with experimental group (CON, TOX-1, TOX-2), week of blood sampling (weeks 3, 10 and 14 of experiment), the interactions between experimental group and week of blood sampling, the parity and the disease status (diseased and veterinary treated or healthy and untreated) as fixed factors. Results obtained from blood samples collected before introducing experimental diets (week 1) were considered as co-variates to account for possible initial differences. A repeated statement was used to consider the frequent measurement on the same cow. Testing various co-variance structures revealed the compound symmetry (CS) as being the most appropriate one according to the Akaike Information Criterion (AICC). Effects were considered to be significant at probabilities ≤0.05, while a trend was assumed for probabilities between 0.05 and 0.1. Results are presented as least square means (LSMEANS) and pooled standard errors of means (PSEM).

In the second data, evaluation step Spearman’s rank correlation coefficients between various variables were estimated to examine the relationships between the blood variables reported in the present paper and further data obtained from the underlying experiment which was formerly evaluated based solely on variance (Winkler et al. 2014a). In order to investigate the relationships between all mentioned variables and the energy metabolism more comprehensively, a multiple regression analysis was additionally performed. Here, those variables closely linked to the energy metabolism were regressed on the intake of net energy lactation (NEL). Based on this regression, the difference between the regressively estimated NEL-intake and the observed NEL intake was defined as residual NEL-intake which is equivalent to that variance proportion which cannot be explained by the regression model. This parameter is frequently used as an efficiency trait and for investigating the unaccounted variance in NEL-intake in relation to other parameters (e.g. Hurley et al. 2016, Potts et al. 2015, Xi et al. 2015).

Finally, to visualize the relationships between the large number of variables in a two-dimensional space, a principal component analysis (PCA) based on correlations was additionally performed.

Correlation and regression analyses were performed using STATISTICA 12.0 (StatSoft, Inc. 2014, Tulsa, Oklahoma, USA).

## Results

### Clinical signs and veterinary treatments

One cow of the CON group died of a peritonitis in the last third of the experiment after a history of treated inappetence, cachexia, phlegmon and arthritis.

Five out of ten cows of each group were treated for metritis, while two, three and three cows of groups CON, TOX-1 and TOX-2 suffered from mastitis, respectively. Other treated illnesses included tylom (1 cow of group TOX-1), phlegmon (1 additional cow of group CON, 1 cow each of groups TOX-1 and TOX-2), abscess (1 cow of group TOX-2) and distortion (1 cow of group TOX-1). Some cows suffered from more than one of the mentioned diseases. Medications included antibiotics, non-steroidal anti-inflammatory drugs, metabolic stimulants, analgesics and spasmolytic drugs.

### Haematology

Generally, all initial values excepting plateletcrit of the white and red blood picture which were measured before the dietary treatments commenced were significantly different (Tables 2 and 3). Total leucocyte and lymphocyte counts significantly decreased over time irrespective of dietary treatments. A similar trend was observed for monocyte counts while neutrophil and eosinophil counts remained constant over the course of the experiment and unaffected by experimental diets. The relative proportion of neutrophils was influenced neither by time nor by dietary treatment. Lymphocyte proportion of group TOX-2 slightly increased over time while those of groups CON and TOX-1 decreased at the same time (*p* = 0.066).

**Table 2:** White blood count of cows fed diets with increasing concentrations of *Fusarium* toxins (LSMEANS, *n* = 10)

*Fusarium* toxins (DON/ZEN, mg/kg at 88 % DM)		Week	Leucocytes [G/l]	Lymphocytes [G/l]	Monocytes [G/l]	Neutrophil granulocytes [G/l]	Eosinophil granulocytes [G/l]	Lymphocytes [%]	Neutrophil granulocytes [%]
CON	0.06/0.02	1	7.9	3.3	0.3	4.0	0.4	41.7	49.3
	0.06/0.02	3	8.2	3.3	0.1	4.0	0.3	42.9	52.0
	0.06/0.02	10	7.9	3.0	0.2	4.5	0.2	37.3	58.0
	0.06/0.02	14	7.5	2.9	0.1	4.1	0.4	39.2	53.8
TOX-1	2.31/0.29	1	8.4	3.4	0.2	4.4	0.4	41.2	51.3
	2.31/0.29	3	7.3	3.3	0.1	3.4	0.4	48.1	43.7
	2.31/0.29	10	7.2	2.7	0.2	3.8	0.4	39.6	51.6
	2.31/0.29	14	7.6	2.7	0.1	4.2	0.4	36.9	55.6
TOX-2	4.61/0.58	1	8.9	3.5	0.6	4.6	0.3	41.0	50.2
	4.61/0.58	3	8.6	3.1	0.1	5.0	0.5	39.3	53.9
	4.61/0.58	10	7.1	3.0	0.1	3.7	0.4	43.5	48.0
	4.61/0.58	14	6.6	2.8	0.1	3.4	0.3	42.8	50.6
*p* values									
DON/ZEN			0.821	0.884	0.565	0.854	0.319	0.860	0.695
Week			*0.035*	***<*** *0.001*	*0.053*	0.726	0.904	0.256	0.548
DON/ZEN x week			0.309	0.608	0.519	0.185	0.863	*0.066*	0.196
Week 1^a^			*<0.001*	*<0.001*	*<0.001*	*0.001*	*0.024*	*<0.001*	*0.007*
Parity^b^			0.199	0.916	0.139	0.208	0.082	0.468	0.624
Diseased^c^			0.622	0.929	0.880	0.405	0.192	0.464	0.300
PSEM			0.6	0.2	0.1	0.5	0.1	2.8	3.9

*LSMEANS* least square means, *PSEM* pooled standard error of means

^a^Effect of the co-variate, i.e. the value measured before experimental diets were introduced;

^b^Cows were classified as primi- and pluriparous cows;

^c^Cows were classified as diseased or not diseased

**Table 3:** Red blood count and platelet characteristics of cows fed diets with increasing concentrations of *Fusarium* toxins (LSMEANS, *n* = 10)

*Fusarium* toxins (DON/ZEN, mg/kg at 88 % DM)		Week	Erythrocytes [T/L]	Haemoglobin [g/dL]	Haematocrit [%]	Mean corpuscular volume [MCV, fL]	Mean corpuscular haemoglobin [MCH, pg]	Mean corpuscular haemoglobin concentration [MCHC, g/dL]	Red cell distribution width [%]	Platelets [G/L]	Plateletcrit [%]	Mean platelet volume [MPV, fL]	Platelet distribution width [%]
CON	0.06/0.02	1	6.3	10.4	34.2	54.9	16.4	30.4	14.8	529	0.17	3.2	17.8
	0.06/0.02	3	5.8	9.5	31.3	54.3	16.3	30.4	14.7	526	0.16	3.0	17.2
	0.06/0.02	10	5.5	9.0	29.6	54.2	16.3	30.6	14.9	536	0.18	3.3	17.2
	0.06/0.02	14	5.6	8.8	30.1	54.1	15.7	29.3	15.5	501	0.17	3.4	17.0
TOX-1	2.31/0.29	1	6.3	10.4	34.8	55.1	16.5	29.8	15.0	525	0.16	3.0	17.8
	2.31/0.29	3	5.8	9.7	31.5	54.0	16.6	30.6	14.6	656	0.18	2.8	17.2
	2.31/0.29	10	5.5	8.7	28.6	52.1	15.9	30.3	14.9	452	0.14	3.1	17.6
	2.31/0.29	14	5.7	9.1	30.0	53.1	16.2	30.2	15.5	518	0.18	3.5	17.8
TOX-2	4.61/0.58	1	6.4	10.3	34.6	54.9	16.4	29.9	15.2	615	0.19	3.1	17.3
	4.61/0.58	3	6.0	10.0	32.3	54.6	16.9	30.9	15.0	681	0.18	2.7	17.8
	4.61/0.58	10	5.9	9.5	31.1	53.5	16.3	30.5	14.6	440	0.13	3.2	17.7
	4.61/0.58	14	5.9	9.6	31.5	54.1	16.4	30.3	15.1	488	0.16	3.4	17.6
*p* values													
DON/ZEN			*0.077*	*0.016*	*0.019*	0.237	0.322	0.352	0.972	0.629	0.467	0.558	0.403
Week			*<0.001*	*<0.001*	*<0.001*	*<0.001*	*0.002*	*0.003*	*<0.001*	**<** *0.001*	*0.057*	*<0.001*	0.793
DON/ZEN x week			0.868	0.513	0.686	0.139	*0.100*	*0.089*	0.102	*0.018*	*0.009*	0.751	0.309
Week 1^a^			*<0.001*	*<0.001*	*<0.001*	*<0.001*	*<0.001*	*0.002*	*<0.001*	0.262	*0.007*	*<0.001*	*<0.001*
Parity^b^			*0.003*	0.598	0.549	0.495	0.138	*0.057*	0.997	0.111	0.479	0.803	*0.069*
Diseased^c^			0.154	0.574	0.430	0.535	0.829	0.330	0.576	0.116	0.337	0.823	0.515
PSEM			0.1	0.2	0.7	0.5	0.2	0.2	0.2	38	0.01	0.1	0.3

*LSMEANS* least square means, *PSEM* pooled standard error of means

^a^Effect of the co-variate, i.e. the value measured before experimental diets were introduced

^b^Cows were classified as primi- and pluriparous cows

^c^Cows were classified as diseased or not diseased

Haemoglobin, haematocrit and erythrocytes significantly decreased over time (Table 3). This time dependent effect occurred at a significantly higher level for group TOX-2 compared to groups CON and TOX-1 for haemoglobin and haematocrit with a similar trend for erythrocyte counts.

Erythrocyte indices mean corpuscular volume (MCV), mean corpuscular haemoglobin (MCH) and mean corpuscular haemoglobin concentration (MCHC) significantly decreased as the experiment progressed. These time effects were slightly modified by dietary treatments for MCHC and MCH as indicated by the trend interactions (*p* = 0.089 and *p* = 0.1, respectively).

Variation in red cell distribution width (RDW) was characterized by an initial constancy followed by a significant increase. Platelet counts of the CON-group showed a less pronounced time-dependent fluctuation than those of groups TOX-1 and TOX-2 resulting in a significant interaction between time and dietary treatment. A similar interactive trend was observed for the plateletcrit (*p* = 0.057). The variation in the width of the platelets (PDW) was influenced neither by time nor by dietary treatments. The mean platelet volume (MPV) first dropped slightly and increased later in the course of the experiment resulting in a significant time effect which was not modified by dietary treatments.

### Flow cytometry

Phenotyping of T cells revealed a significant interaction between time and dietary treatment for the proportion of CD4+ cells caused by a drop in cows of group TOX-2 at the end of the experiment, compared to the other groups which maintained a constant CD4+ cell proportion (Table 4). The proportion of CD8+ cells decreased in groups TOX-1 and TOX-2, while an increase was found for the CON-group which caused the significant interaction between time and dietary treatment. The CD4+/CD8+ ratio tended to decrease over time (*p* = 0.098), while absolute counts of CD4+ and CD8+ cells remained unaffected by time and dietary treatments.

**Table 4:** Relative proportions of CD4+ and CD8+ cells of total lymphocytes and total counts in peripheral systemic blood of cows fed diets with increasing concentrations of *Fusarium* toxins (LSMEANS, *n* = 10)

*Fusarium* toxins (DON/ZEN, mg/kg at 88 % DM)		Week	CD4+ [%]	CD8+ [%]	CD4+/CD8+	CD4+ [G/l]	CD8+ [G/l]
CON	0.06/0.02	3	25.0	11.3	3.8	0.9	0.4
	0.06/0.02	10	25.8	12.3	2.2	0.8	0.5
	0.06/0.02	14	28.5	15.7	1.8	1.0	0.5
TOX-1	2.31/0.29	3	25.9	12.0	2.3	0.9	0.4
	2.31/0.29	10	30.3	12.9	2.5	0.9	0.4
	2.31/0.29	14	26.7	11.2	2.1	0.7	0.3
TOX-2	4.61/0.58	3	23.9	12.1	3.1	0.9	0.5
	4.61/0.58	10	27.9	13.9	2.0	1.1	0.6
	4.61/0.58	14	16.2	9.9	1.8	1.0	0.5
*p* values							
DON/ZEN			*0.020*	0.793	0.888	0.650	0.110
Week			*0.061*	0.457	*0.098*	0.827	0.866
DON/ZEN *x* week			*0.020*	*0.043*	0.632	0.146	0.720
Parity^a^			0.513	0.772	0.346	0.581	0.750
Diseased^b^			*0.051*	0.869	0.656	0.955	0.902
PSEM			2.2	1.7	0.8	0.1	0.5

*LSMEANS* least square means, *PSEM* pooled standard error of means

^a^Cows were classified as primi- and pluriparous cows

^b^Cows were classified as diseased or not diseased

Reactive oxygen species (ROS) formation of peripheral blood mononuclear cells (PBMC) and of total granulocytes were only slightly influenced by experimental factors (Table 5). ROS+ PBMC mean fluorescence intensity (MFI) tended to decrease from week 4 to week 8 of the experiment (*p* = 0.066). The proportion of TPA-stimulated ROS+ granulocytes converged to a similar level for all experimental groups at week 8 starting from distinct differences between groups at week 4 (*p* = 0.053). The MFI of TPA-stimulated ROS+ granulocytes and the stimulation index (SI) remained constant or slightly increased in groups TOX-1 and TOX-2 while a decrease was evident for Group CON (*p* = 0.069 and *p* = 0.053, respectively).

**Table 5:** Reactive oxygen species (ROS) formation of peripheral blood mononuclear cells (PBMC) and of total granulocytes (GR) and ability of GR to respond to a stimulus ex vivo. Blood samples were collected from cows fed diets with increasing concentrations of *Fusarium* toxins (LSMEANS, *n* = 10)

*Fusarium* toxins (DON/ZEN, mg/kg at 88 % DM)		Week	ROS formation							GR stimulation index ^a^
			Basal (unstimulated)					TPA-stimulated		
			ROS+ GR [MFI]	ROS+ GR [%]	ROS+ GR [G/l]^b^	ROS+ PBMC [MFI]	ROS+ PBMC [%]	ROS+ GR [MFI]	ROS+ GR [%]	
CON	0.06/0.02	10	6939	5.4	0.22	5108	2.3	62,635	100.0	9.0
	0.06/0.02	14	8221	9.2	0.34	3447	0.6	46,746	96.6	6.3
TOX-1	2.31/0.29	10	8051	7.4	0.26	4375	0.3	53,030	93.0	7.1
	2.31/0.29	14	6768	8.1	0.36	4322	3.5	56,466	99.0	10.4
TOX-2	4.61/0.58	10	8089	12.3	0.50	4475	0.5	57,898	97.6	8.1
	4.61/0.58	14	7261	11.6	0.47	3564	2.3	57,607	97.7	9.7
*p* values										
DON/ZEN			0.970	0.227	0.150	0.150	0.953	0.622	0.372	0.592
Week			0.581	0.425	0.363	0.363	0.406	0.196	0.569	0.470
DON/ZEN *x* week			0.109	0.537	0.613	0.613	0.335	*0.053*	*0.069*	*0.053*
Parity^c^			0.147	0.170	0.447	0.447	0.676	*0.081*	0.195	0.802
Diseased^d^			0.886	0.800	0.903	0.903	0.927	0.702	0.245	0.644
PSEM			888	2.5	0.10	0.10	1.8	3770	1.8	1.2

Further abbreviations: *LSMEANS* least square means, *TPA* tetradecanoyl-12,13-phorbol acetate, *MFI* mean fluorescence intensity, arbitrary units, *ROS+* ROS positive cells, *PSEM* pooled standard error of means

^a^Ratio between the MFI of TPA-stimulated and unstimulated ROS+ GR

^b^Peripheral blood granulocytes exhibiting a basal ROS production (total blood granulocytes multiplied with the proportion of basal ROS+ GR)

^c^Cows were classified as primi- and pluriparous cows

^d^Cows were classified as diseased or not diseased

### Clinical chemistry

Excepting albumin and GLDH, all other blood clinical-chemical variables were significantly different at the beginning of the experiment (Table 6). Moreover, all parameters underwent a significant longitudinal pattern with the exception of total protein while dietary treatment did not influence any of the variables excepting BHB which started to decrease later in Group TOX-2 causing the significant interaction between time and dietary treatment. An opposite interactive trend was observed for the glucose concentration which failed to increase in group TOX-2 as the experiment progressed (*p* = 0.081).

**Table 6:** Clinical–chemical characteristics of cows fed diets with increasing concentrations of *Fusarium* toxins (LSMEANS, *n* = 10)

*Fusarium* toxins (DON/ZEN, mg/kg at 88 % DM)		Week	Glucose [mg/dL]	BHB [mMol/L]	NEFA [mMol/L]	Cholesterol [mg/dL]	Total triglycerides [mg/dL]	Albumin [g/l]	Total protein [g/l]	Urea [mg/dL]	Total bilirubin [mg/dL]	ASAT [U/l]	GGT [U/l]	GLDH [U/l]
CON	0.06/0.02	1	50.4	1.2	0.9	97.7	13.8	36.0	73.0	25.1	1.6	103.3	21.6	12.3
	0.06/0.02	3	47.7	1.2	0.7	109.0	20.2	33.8	70.7	23.5	1.6	86.0	27.6	18.3
	0.06/0.02	10	54.8	0.7	0.5	206.1	14.3	36.7	77.1	22.5	1.5	69.6	26.5	23.2
	0.06/0.02	14	49.0	0.7	0.2	199.8	19.9	32.4	71.8	33.0	1.5	73.7	30.6	33.7
TOX-1	2.31/0.29	1	48.1	1.3	0.9	96.7	14.0	35.7	72.2	22.1	1.6	104.4	21.0	11.6
	2.31/0.29	3	47.6	1.3	0.9	105.5	23.0	34.2	72.0	25.2	1.6	97.5	29.0	22.3
	2.31/0.29	10	60.6	0.6	0.4	204.9	17.5	35.4	75.1	23.5	1.5	59.4	27.2	17.1
	2.31/0.29	14	55.1	0.6	0.2	195.3	20.0	34.3	76.6	28.6	1.5	77.4	39.1	37.4
TOX-2	4.61/0.58	1	52.0	1.1	0.9	94.8	13.3	36.1	75.5	22.3	1.6	105.9	22.1	14.0
	4.61/0.58	3	45.0	0.9	0.7	123.8	22.7	34.0	73.4	23.7	1.6	88.7	27.8	20.6
	4.61/0.58	10	48.4	1.2	0.2	177.9	11.2	34.9	69.0	23.4	1.5	73.1	22.8	67.8
	4.61/0.58	14	47.9	0.7	0.2	193.2	23.0	34.1	72.6	31.3	1.5	81.3	38.8	58.0
*p* values														
DON/ZEN			0.151	0.927	0.412	0.847	0.292	0.975	0.767	0.638	0.561	0.742	0.512	0.217
Week			*0.004*	*<0.001*	*<0.001*	*<0.001*	*<0.001*	*0.007*	0.766	*<0.001*	*<0.001*	*<0.001*	*<0.001*	*0.027*
DON/ZEN *x* week			*0.081*	*0.001*	0.389	0.369	0.100	0.677	0.128	0.390	0.748	0.703	0.405	0.484
Week 1^a^			*<0.001*	*0.001*	*0.001*	*0.001*	*0.003*	*0.059*	*0.011*	*0.005*	*<0.001*	*<0.001*	*0.001*	0.171
Parity^b^			0.909	0.212	0.467	0.333	*0.006*	*0.043*	0.429	*0.096*	0.400	0.228	0.744	0.791
Diseased^c^			0.569	0.207	0.378	0.272	0.206	0.182	0.108	0.494	0.863	0.553	0.642	*0.067*
PSEM			2.7	0.1	0.1	11.1	1.5	1.0	2.4	1.5	0.0	6.9	2.8	14.2

Further abbreviations: *BHB* 3-β-hydroxybutyrate, *NEFA* non-esterified fatty acids, *GGT* Gamma-glutamyl transferase, *ASAT* aspartate-aminotransferase, *GLDH* glutamate-dehydrogenase, *LSMEANS* least square means, *PSEM* pooled standard error of means

^a^Effect of the co-variate, i.e. the value measured before experimental diets were introduced

^b^Cows were classified as primi- and pluriparous cows

^c^Cows were classified as diseased or not diseased

The significant time effects of the other parameters included a decrease in NEFA, total bilirubin, albumin and ASAT; while for triglycerides, urea, GGT and GLDH an increase was noticed. Besides, the pronounced time effects, parity influenced triglyceride and albumin concentration which were significantly higher and lower for the heifers compared to the pluriparous cows.

### Estimation of NEL intake and residual NEL intake

Based on a gradual multiple linear regression approach, only those variables were included which were suggested to represent so-called energy sinks explaining variance in the observed net energy lactation intake (NEL-in_obs_) (Hurley et al. 2016, Potts et al. 2015, Xi et al. 2015) and which were proven to contribute significantly to the regression. Therefore, energy corrected milk (ECM), days in milk (DIM), metabolic body weight (b.w.), non-esterified fatty acids (NEFA) and 3-β-hydroxybutyrate (BHB) were used for regression analysis. Based on fitted regression coefficients, NEL intake was estimated (NEL-in_pred_) as follows:

$$ \begin{array}{l}NEL-{\mathrm{in}}_{\mathrm{pred}}=-4.79+1.{88}^{***}\cdot ECM+0.{53}^{*}\cdot \mathrm{b}.\mathrm{w}.{\left(\mathrm{kg}\right)}^{0.75}+0.{5}^{***}\cdot \mathrm{DIM}-14.0{2}^{*}\cdot \mathrm{NEFA}-8.{84}^{*}\cdot BHB\hfill \\ {}\left[{r}^2=0.70{9}^{***};\kern0.5em \mathrm{residual}\kern0.5em \mathrm{standard}\kern0.5em \mathrm{deviation}=16\kern0.5em MJ\kern0.5em NEL/\mathrm{d}{;}^{*}\kern0.5em p<0.05,{\kern0.5em }^{***}p<\kern0.5em 0.001\right]\hfill \end{array} $$

Based on the coefficient of determination (*r* ^2^) of 0.709, it can be deduced that approximately 71 % of the variance in NEL intake could be explained by the regression while 29 % remained unexplained and corresponded to a residual standard deviation of 16 MJ NEL/d. Based on the difference between the observed and the estimated NEL intake, the residual NEL intake (RSEI) was calculated as follows:

$$ \mathrm{RSEI}=NEL\hbox{-} {\mathrm{in}}_{obs}\hbox{-} NEL\hbox{-} {\mathrm{in}}_{\mathrm{pred}} $$

**Fig. 1:**
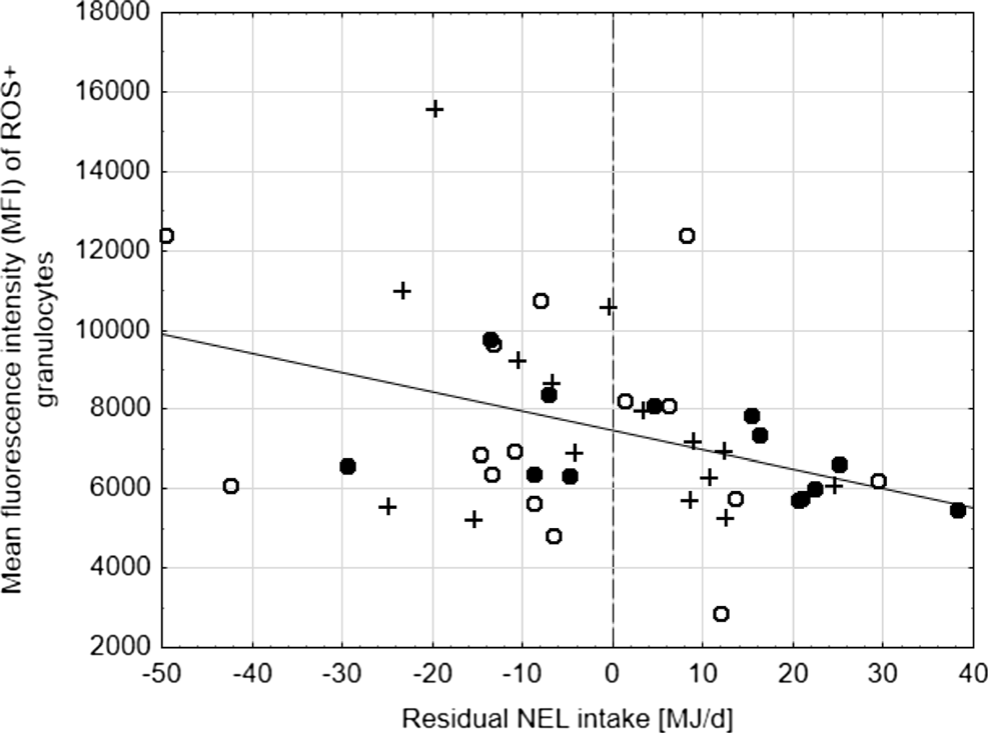
Relationship between residual NEL intake and mean fluorescence intensity (MFI) of ROS+ granulocytes: *y* = 7459–48.8·*x*, *r* ^*2*^ = 0.148, residual standard deviation = 1435 MFI (*p* values for regression parameters and *r* ^2^ < 0.05); *black circle*, group CON; *white circle*, group TOX-1; *plus sign*, group TOX-2

Correlation analysis demonstrated that only the blood urea concentration correlated positively with RSEI (*r* = 0.322) while the mean fluorescence intensity (MFI) of the ROS-positive granulocytes suggested a negative relationship (*r* = −0.385) which was further elaborated to a linear regression (Fig. 1).

### Principal component analysis (PCA)

A principal component analysis (PCA) was performed to visualize possible relationships between 46 variables in different units. Therefore, the PCA was based on correlations rather than on co-variances. The results revealed that the first two components (PC 1 and PC 2) extracted approximately 26 % of the total variance. The scree plot as a visualization between successively extracted components and the corresponding eigenvalues did not show a distinct break point which is accepted to indicate the transition from the most important components to those not contributing significantly to the total explained variance. Here, the eigenvalue of 1.0 as the mean value of all 46 eigenvalues corresponded to a total of 15 extracted components which explained approximately 81 % of the total variance.

**Fig. 2:**
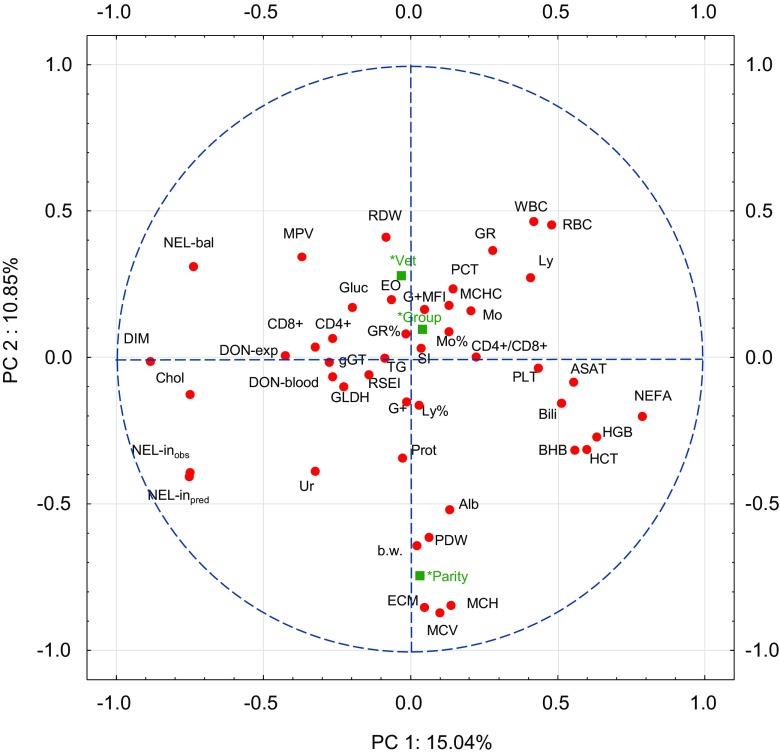
Principal Component Analysis for a two-dimensional visualization of the relationships between 46 variables collected from the experiment: *Variables for the analysis*: *WBC* white blood cell count, *LY%* lymphocyte proportion, *MO%* monocyte proportion, *GR%* neutrophil granulocyte proportion, *LY* lymphocyte count, *MO* monocyte count, *GR* neutrophil granulocyte count, *EO* eosinophil granulocyte count, *RBC* red blood cell count, *HGB* haemoglobin, *HCT* haematocrit, *MCV* mean corpuscular volume, *MCH* mean corpuscular haemoglobin, *MCHC* mean corpuscular haemoglobin concentration, *RDW* red cell distribution width, *PLT* platelet count, *PCT* plateletcrit, *MPV* mean platelet volume, *PDW* platelet distribution width, *Alb* albumin, *Gluc* glucose, *Chol* cholesterol, *ASAT* aspartate-aminotransferase, *gGT* gamma-glutamyl transferase, *Bilirubin* bilirubin, *GLDH* glutamate-dehydrogenase, *Prot* protein, *TG* triglycerides, *Ur* urea, *BHB* 3-β-hydroxybutyrate, *NEFA* non-esterified fatty acids, *CD4* **+** CD4+ cells of total lymphocytes, *CD8* **+** CD8+ cells of total lymphocytes, *CD4* **+/** *CD8* **+** ratio between CD4+ and CD8+ cells, **G+** Reactive oxygen species (ROS) positive granulocytes, **G +** *MFI* mean fluorescence intensity (MFI) of ROS+ granulocytes, *SI* Ratio between the MFI of TPA (tetradecanoyl-12,13-phorbol acetate)-stimulated and unstimulated ROS+ GR, *DON-exp* **.** daily deoxynivalenol (DON) exposure, *DON* **-** *blood* DON and de-epoxy-DON concentration in blood, *DIM* days in milk, *ECM* energy corrected milk, *NEL-in*_obs_ daily intake of net energy lactation (NEL), *NEL-in*_pred_ estimated NEL intake, *RSEI* residual net energy intake (NEL-in_obs_-NEL-in_pred_), *NEL* **-** *bal* **.** NEL balance, *b* **.** *w* **.** metabolic body weight. *Additional variables* : * *Group* experimental group, * *Vet* disease/veterinary treatment status, * *Parity* lactation/parity number

Plotting the correlations between all 46 variables and PC 1 against the corresponding correlations to PC 2 revealed relatively poor correlations of many variables both to PC 1 and PC 2 as indicated by their localization close to the centre of the cross in Fig. 2. The closer the variables to the outer circle, the closer the correlation of a particular variable either to PC 1 or PC 2 or to both. While energy corrected milk (ECM) and the erythrocyte indices mean corpuscular volume (MCV) and mean corpuscular haemoglobin (MCH) were highly negatively correlated to PC 2 but not to PC 1, the opposite was observed for the days in milk (DIM) and the blood cholesterol concentration. Positive correlations to PC 1 were observed for non-esterified fatty acids (NEFA), haemoglobin (HGB), haematocrit (HCT) and 3-β-hydroxybutyrate (BHB); these variables correlated weakly negative to PC 2 at the same time. Projecting the additional variables into the variable space showed that neither the experimental group (group) nor the disease/treatment status (Vet) of the cows were related to PC 1 or PC 2, while the parity clustered together with ECM, MCH and MCV (Fig. 2).

## Discussion

Ruminants in general and dairy cows in particular are regarded as quite resistant to the presence of DON and ZEN commonly found in feedstuffs mainly due to ruminal toxin metabolism (e.g. Dänicke and Brezina 2013, Dänicke and Winkler 2015). The guidance values for critical dietary concentrations of DON and ZEN as recommended by the European Commission (European Commission 2006) were derived from extensive literature compilations performed by European Food Safety Authority (European Food Safety Authority 2004a, 2004b). However, due to the limited data base nearly exclusively performance parameters such as DM-intake (DMI) and milk yield were used as toxicological endpoints.

Besides these gross parameters, health-associated traits might be influenced by feeding *Fusarium*-toxin contaminated feed without compromising general performance of the cows (e.g. Keese et al. 2008a, Kinoshita et al. 2015, Korosteleva et al. 2007, Korosteleva et al. 2009). Therefore, we not only completed the recently reported effects of increasing dietary concentrations of DON and ZEN on performance of the early lactating cow (Winkler et al. 2014a) with health-related haematological, clinical–chemical and immunological data, but we also tried to integrate all these data to obtain a more holistic view of DON and ZEN effects on cows.

Clinical-chemical traits showed the typical course which can be observed in cows during the first weeks after parturition. Therefore, glucose concentration increased with time while BHB and NEFA decreased at the same time indicating the transition from extensive *post partum* lipolysis to a positive energy balance. Looking closer to these general time-dependent changes, it appeared that feeding the TOX-2 diet resulted in a modified kinetics since glucose increase occurred at a lower level or was even absent when compared to the other groups (Table 6). This was accompanied by a BHB peak during week 10 *post partum* which was absent in the other groups indicating a compromised hepatic BHB-utilization since NEFA, as the precursors were not elevated at the same time. A ruminally butyrate driven BHB flow to the liver can probably be excluded since DMI of this group did not differ from the other groups (Winkler et al. 2014a). If a reduced hepatic BHB utilization is assumed, then this was not associated with a treatment-related general compromising of liver function and hepatocyte integrity as indicated by unaltered albumin, triglyceride, cholesterol, total bilirubin and urea concentration, as well as unchanged serum activities of ASAT, GLDH and GGT, respectively. Thus, ketogenesis might be modified at the cellular level after feeding the ration with the highest DON and ZEN concentration. In contrast to the present results, blood urea, protein and globulin were found to be increased in mid-lactating cows after feeding a contaminated TMR containing 3.2 mg DON and 0.24 mg ZEN/kg (Korosteleva et al. 2007).

Regarding the haemogram, the most striking treatment effects were the increased erythrocyte counts, haematocrit and haemoglobin concentration. Due to similar directed alterations, the erythrocyte indices remained unaffected by treatments. Taking the progression in time into consideration, it appeared that the general time-dependent decrease occurred at a higher level in group TOX-2 compared to the other groups. It needs to be stressed that these significant treatment effects occurred within the reference ranges which are given at 5–10 T/L for erythrocytes, 28–38 % for haematocrit, and 9–14 g/dLfor haemoglobin (Moritz 2014). DON related increases in erythrocyte counts and platelets have been reported in pigs (Prelusky et al. 1994) and were discussed as either myelotoxic effects or just being the result of a haemo-concentration due to an altered drinking behaviour or disturbances in the body water balance. Water intake was not evaluated in the present study. Although other blood cells and clinical-chemical traits did not show a similar haemo-concentrating behaviour as erythrocytes, toxin related alterations in blood volume cannot be excluded. Interestingly, Korosteleva et al. (2007) reported a hypernatraemia and an associated hyperosmolality in mid-lactating cows fed mainly with DON-contaminated TMRs (3.2–3.5 mg DON/kg) relative to the control groups and attributed these effects to a subclinical reduction in water intake.

Mechanistically, myelotoxic effects due to DON are rather impossible in the view that DON is largely metabolized to de-DON pre-systemically by rumen microbes (for review see Dänicke and Brezina 2013). This metabolite was characterized as much lower cytotoxic than its parent compound DON in bovine PBMC (Dänicke et al. 2011). In contrast, in pigs, only traces of de-DON can be detected in the circulation while the unmetabolized DON comprises the major compound (for review see Dänicke and Brezina 2013). In the present experiment, neither red cell nor platelet distribution width was altered due to treatments further supporting the view that toxic effects on bone marrow probably did not occur. However, it needs to be stressed that the contaminated maize grain used in the present experiment originated from an artificial *Fusarium graminearum* inoculation as described by Rempe et al. (2013). It contained not only DON (37,540 μg/kg) and ZEN (4269 μg/kg) but also their modified forms such as DON-3-glucoside (4426 μg/kg), 3- and 15-acetyl-DON (545 and 3657 μg/kg), ZEN-4-sulphate (183 μg/kg) but other toxins like butenolid (2714 μg/kg), aurofusarin (25,904 μg/kg), culmorin (13,625 μg/kg), 15- and 5-OH-culmorin (2350 and 12,081 μg/kg). While modified forms of DON and ZEN might be regarded as DON and ZEN equivalents due to ruminal metabolism, the knowledge about the ruminal metabolism and the toxic effects of the other toxins in the bovine is rather scarce. From this perspective, bone marrow effects of co-contaminants cannot be excluded.

CD4+/CD8+ ratio is regarded as critical in maintaining T cell homeostasis and immune regulation in cows (Mehrzad and Zhao 2008). Based on own results and on literature compilation, Mehrzad and Zhao (2008) considered a ratio of ~2.5 as physio-immunologically normal. A CD4+/CD8+ ratio of ~2.5 corresponded to a higher lymphoproliferative response of T cells in primiparous cows compared to pluriparous cows which showed a ratio of approximately 4.0 which was paralleled by a lower T cell proliferation. However, it needs to be stressed that cows in that study were in mid-lactation, while cows of the present experiment were at the beginning of the lactation period. Here, the CD4+/CD8+ ratio started with values between 2.1 up to 3.8 and decreased as experiment progressed to values between 1.8 and 2.1. Thus, early lactating cows seem to be in an immunologically more dysregulated T cell balance compared to later stages of lactation. This finding is in general agreement with recent findings in transition cows (Schulz et al. 2015, Tienken et al. 2015) but contrasts another experiment where time and treatment (body condition score, monensin, essential oils) around calving influenced the CD4+/CD8+ ratio in an interactive manner (Drong et al. 2016). A successive increase in dietary energy concentration resulted in a significant decrease of the CD4+/CD8+ ratio from 2.5 to 2.1 in non-gravid and non-lactating cows (Dänicke et al. 2016). In the present experiment, the CD4+/CD8+ ratio remained uninfluenced by dietary treatment although significant interactions between treatment and time were observed for the percentages of CD4+ and CD8+ cells. However, as the time dependent decrease in the proportions of both T cell populations was observed only in Group TOX-2 at the same time, the resulting ratio was not influenced and caused the significant interactions between time and treatment. Therefore, the mechanisms influencing T cells at higher dietary exposure to *Fusarium* toxins seem to be different from those affecting specific T cell populations. The fact that both CD4+ and CD8+ cells were influenced in a similar way might hint at direct toxic effects. Interestingly, exposure of dairy cows to a *Fusarium* toxin contaminated diet containing mainly DON (4.6 mg/kg DM) resulted in an ex vivo PBMC viability decrease by 18 %, although maximum serum de-DON and DON levels of 52 ng/ml (0.19 μM) and 9 ng/ml (0.03 μM), respectively, were much lower than the corresponding cytotoxic in vitro concentrations of >18.3 μM for de-DON and 0.5 μM for DON (Dänicke et al. 2011). Authors hypothesised that other treatment effects might have influenced the PBMC viability indirectly such as varying energy and nutrient supply mediated by treatment related differences in DM or NEL intake.

In the present experiment, neither the proportions of CD4+ nor of CD8+ cells formed a distinct cluster with milk yield (ECM) nor NEL-intake but with dietary DON exposure (as an indicator for the exposure to all *Fusarium* toxins present in the diets) and DON and de-DON residue levels in blood. Albeit only weakly correlated to PC 1 the Pearson correlation coefficients between CD4+ cells, DON exposure and DON and de- DON residue levels in blood amounted to −0.35 (*p* < 0.05) and −0.43 (*p* < 0.05), respectively, supporting the view that the lower proportion of the T cell type was indeed associated with toxin exposure and not with DM or NEL intake. The corresponding correlations between CD8+ cell proportions suggested similar directions but failed to reach significance; −0.26 (*p* > 0.05) for DON exposure and −0.17 (*p* > 0.05) for DON and de-DON residue levels in blood. As total leucocyte counts were not influenced by increasing dietary *Fusarium* toxin contamination but the proportions of total lymphocytes increased at the same time, the treatment related decreases in the proportions of CD4+ and CD8+ cells were counterbalanced from a quantitative viewpoint. Therefore, similar concentrations of CD4+ and CD8+ cell counts were present in the blood irrespective of treatment group.

RSEI as an indicator for variance of NEL intake not explainable by the common energy sinks correlated negatively with the MFI of the ROS-positive granulocytes suggesting that cows consuming more energy than the average of all experimental animals across the longitudinal axis were characterized by a less pronounced basal intensity of granulocytes to mount an oxidative burst. Moreover, cows consuming less energy than the average of the herd appeared to exhibit a more variable basal ROS-forming activity. All these effects occurred independently of exposure to *Fusarium*-toxins but seem to indicate a general inverse relationship between energy (NEL) consumption and basal granulocyte function. On the other hand, it needs to be considered that a negative RSEI more frequently occurred at the onset of lactation in a period of a negative energy balance. Therefore, the higher MFI of ROS+ granulocytes at lower RSEI coincides with a more pronounced negative energy balance and could hint at the more pro-inflammatory situation of the cows during this period.

Looking closer to the results of the PCA, all variables which were considered as energy sinks for estimating NEL intake and subsequently RSEI, namely ECM, DIM, metabolic body weight, serum NEFA and BHB concentrations demonstrated reasonable correlations to PC 1 and/or PC 2 while the resulting RSEI did not. On the other hand RSEI and basal MFI of ROS+ granulocytes clustered together with other parameters close to the origin of the cross of the unit circle suggesting independency of PC 1 and PC 2 and supporting the view that the performed PCA failed to identify distinct clusters of variables explaining treatment–related effects. This makes clear that the discussed significant treatment effects for some parameters can be regarded as more or less isolated pathophysiological events which were not strongly related to the majority of the uninfluenced parameters.

## Conclusions

The decrease in the CD4+ cell proportion in peripheral blood along with the unchanged concentrations of this cell type in blood of cows fed with the highest dietary concentration of *Fusarium* toxins indicates qualitative effects rather than quantitative consequences for the balance of CD4+ and CD8+ cells. Moreover, the striking haemo-concentrating effects observed for haematocrit, erythrocytes and haemoglobin due to feeding diet TOX-2 hint at regulatory mechanisms in water and electrolyte balance and/or at the level of bone marrow.

Therefore, investigations are necessary which specifically address the effects of the *Fusarium* toxins DON, ZEN and other associated toxins on mechanisms underlying the observed haematological alterations. In doing so, the investigation of immunological responses under standardized immuno-stimulatory conditions (e.g. by injection of endotoxins) could be a helpful tool when DON/ZEN contaminated diets are fed at the same time.
